# INDEL variation in the regulatory region of the major flowering time gene *LanFTc1* is associated with vernalization response and flowering time in narrow‐leafed lupin (*Lupinus angustifolius* L.)

**DOI:** 10.1111/pce.13320

**Published:** 2018-05-23

**Authors:** Candy M. Taylor, Lars G. Kamphuis, Weilu Zhang, Gagan Garg, Jens D. Berger, Mahsa Mousavi‐Derazmahalleh, Philipp E. Bayer, David Edwards, Karam B. Singh, Wallace A. Cowling, Matthew N. Nelson

**Affiliations:** ^1^ UWA School of Agriculture and Environment The University of Western Australia Perth Western Australia 6009 Australia; ^2^ Agriculture and Food Commonwealth Scientific and Industrial Research Organisation Floreat Western Australia 6014 Australia; ^3^ Centre for Crop and Disease Management Curtin University Bentley Western Australia 6102 Australia; ^4^ School of Biological Sciences The University of Western Australia Perth Western Australia 6009 Australia; ^5^ The UWA Institute of Agriculture The University of Western Australia Perth Western Australia 6009 Australia; ^6^ Natural Capital and Plant Health Royal Botanic Gardens, Kew Ardingly West Sussex RH17 6TN UK

**Keywords:** *cis*‐regulation, insertion/deletion (INDEL), variant series, *FLOWERING LOCUS T* (*FT*)

## Abstract

Narrow‐leafed lupin (*Lupinus angustifolius* L.) cultivation was transformed by 2 dominant vernalization‐insensitive, early flowering time loci known as *Ku* and *Julius* (*Jul*), which allowed expansion into shorter season environments. However, reliance on these loci has limited genetic and phenotypic diversity for environmental adaptation in cultivated lupin. We recently predicted that a 1,423‐bp deletion in the *cis*‐regulatory region of *LanFTc1*, a *FLOWERING LOCUS T* (*FT*) homologue, derepressed expression of *LanFTc1* and was the underlying cause of the *Ku* phenotype. Here, we surveyed diverse germplasm for *LanFTc1 cis*‐regulatory variation and identified 2 further deletions of 1,208 and 5,162 bp in the 5' regulatory region, which overlap the 1,423‐bp deletion. Additionally, we confirmed that no other polymorphisms were perfectly associated with vernalization responsiveness. Phenotyping and gene expression analyses revealed that *Jul* accessions possessed the 5,162‐bp deletion and that the *Jul* and *Ku* deletions were equally capable of removing vernalization requirement and up‐regulating gene expression. The 1,208‐bp deletion was associated with intermediate phenology, vernalization responsiveness, and gene expression and therefore may be useful for expanding agronomic adaptation of lupin. This insertion/deletion series may also help resolve how the vernalization response is mediated at the molecular level in legumes.

## Abstract 2

“Greater genetic and phenotypic diversity for flowering time would enhance the adaptation of narrow‐leafed lupin to Australian and northern European agricultural environments. We studied the major floral integrator gene in narrow‐leafed lupin, *LanFTc1*, and found a series of three overlapping independent deletions within the promoter region that reduce vernalization responsiveness, shorten flowering time, and heighten expression of *LanFTc1*. The smallest deletion results in a unique intermediate phenotype, which may be valuable for breeding purposes and expanding the agronomic adaptation of lupin to new and existing production regions. Additionally, the insertion/deletion series may also help to increase knowledge of how signalling within the vernalization pathway is mediated at a molecular level in legumes.”

## 1 INTRODUCTION

Narrow‐leafed lupin (*Lupinus angustifolius* L.) is one of three fully domesticated Old World *Lupinus* species originating from the Mediterranean and northern Africa (Gladstones, 1974). It is predominantly grown in Australia and several northern European countries, including Poland, Russia, Germany, Ukraine, and Belarus, as a winter and summer annual pulse crop, respectively (FAO, 2014; Gladstones, 1970). The grain is most commonly marketed as a high‐protein livestock and aquaculture feed. However, in light of the nutritional and metabolomics properties of narrow‐leafed lupin seed (Lima‐Cabello et al., 2017) and the associated benefits to human health and disease prevention (Foyer et al., 2016; Kouris‐Blazos & Belski, 2016), it is also being promoted in the human food market. In addition to high‐protein grain production, narrow‐leafed lupin has great agricultural value as a superior break crop. Lupin crops mobilize soil‐bound phosphorus through carboxylate exudation (Lambers, Clements, & Nelson, 2013) and improve soil nitrogen through symbiosis, which, together with disease and weed control, is beneficial to the performance of subsequent crop rotations (Seymour, Kirkegaard, Peoples, White, & French, 2012).

Following domestication, one of the most important achievements in narrow‐leafed lupin breeding has been the manipulation of phenology. In most wild populations, a prolonged period of exposure to cold winter temperatures, known as vernalization, is required to promote the transition from vegetative to reproductive growth (Rahman & Gladstones, 1972). However, this trait caused great difficulty in the global transitioning of the species from a green manure and fodder crop into a broad acre grain crop. Within key production zones of Australia, winter temperatures are often mild and incapable of reliably saturating the vernalization requirement from year to year, leading to delayed phenology, susceptibility to terminal drought stress and reduced final yields (Berger, Buirchell, Luckett, Palta, et al., 2012; Gladstones, 1977). Similarly, strong vernalization requirement was also a problem for summer cropping of lupin in northern Europe, where cool and wet autumn conditions hindered grain maturation, particularly if sowing was delayed by the late arrival of spring or growing regions receive high amounts of summer rainfall (Kubok, 1988; Mikołajczyk, 1966). Two naturally occurring dominant mutations were discovered independently during the 1960s in Australia and northern Europe and effectively removed the requirement for vernalization, thereby improving the adaptation and yield stability of narrow‐leafed lupin in short season environments. The most widely adopted of these is *Ku*, which arose as a spontaneous mutant in the cultivar “Borre” and is capable of advancing flowering time by up to 5 weeks in Australia (Gladstones & Hill, 1969). It has been selected in almost all elite Australian cultivars released since the 1970s (Cowling, 1999; Stefanova & Buirchell, 2010) and has also been commonly used in European breeding programs (Boersma, Buirchell, Sivasithamparam, & Yang, 2007). The second mutation, *Julius* (*Jul*), was first discovered in the Russian bred cultivar, Krasnolistny, and became an important source of early phenology in Polish breeding during the 1980s (Kubok, 1988; Mikołajczyk, 1966). Due to striking similarity in photoperiodic as well as vernalization responsiveness, *Ku* and *Jul* were thought to be controlled by the same gene (Rahman & Gladstones, 1972).

Global production of narrow‐leafed lupin grain has stagnated in recent years with a leading cause being the limited genetic and adaptive diversity available within domesticated breeding pools of narrow‐leafed lupin (Berger, Buirchell, Luckett, & Nelson, 2012). This lack of diversity largely stems from the species' recent domestication, which was based on a small number of founding individuals, and subsequent strong and persistent selection for key traits, such as phenology, all of which have resulted in severe genetic bottlenecks (Berger, Buirchell, Luckett, & Nelson, 2012; Cowling, 1999; Stefanova & Buirchell, 2010). Among the consequences for these bottlenecks is a reduction in genetic variation for flowering time within a domestic background. Furthermore, any variation that is present is hidden by the dominant overriding effect of the *Ku* and *Jul* loci.

A lack of phenological diversity is problematic for two main reasons. First, despite the profound influence *Ku* and *Jul* have had in adapting narrow‐leafed lupin to short‐season production environments, many regions still lack options for cultivars adequately matched to their respective climates (Berger, Buirchell, et al., 2008; Berger, Buirchell, Luckett, Palta, et al., 2012). The southwest of Western Australia and the eastern states of Australia are two such regions, where longer growing seasons coupled with reduced evapotranspiration could support later flowering cultivars and achieve higher yields (C. Chen, Fletcher, Lawes, Berger, & Roberston, 2017). Second, the reliance on *Ku* and lack of genetic diversity due to domestication bottlenecks will reduce the ability of breeders to select stress tolerant cultivars adapted to climate change, which threatens future yield potential of lupins and crop harvestability (Nelson, Berger, & Erskine, 2010). New genetic variation for phenology is required to increase the adoption of narrow‐leafed lupin in longer season environments and to maintain or extend feasible production zones in the face of climate change.

Recently, the genetic identify of *Ku* was revealed as a *FLOWERING LOCUS T* (*FT*) homologue, *LanFTc1* (Nelson et al., 2017). In *Arabidopsis*, *FT* has a well‐defined role as a floral integrator gene, coordinating signals from the vernalization, photoperiod, and circadian clock pathways to promote flowering at an opportune time (Turck, Fornara, & Coupland, 2008). A 1,423‐bp deletion in the promoter region of *LanFTc1* was implicated as the casual mutation for the *Ku* allele, as its presence was perfectly predictive of vernalization responsiveness in 216 wild and domesticated accessions and was associated with derepressed expression of *LanFTc1* in the absence of vernalization (Nelson et al., 2017). Given the demonstrated capacity for mutations in the noncoding sequence of this gene to modify its expression and plant phenology, we endeavoured to find other polymorphisms that may affect *cis*‐regulation of *LanFTc1* and provide alternative sources of flowering time variation. Here, we report the discovery of a series of insertion/deletions (INDELs) in the 5' regulatory region of this gene, which are associated with altered vernalization responsiveness, flowering time, and *LanFTc1* gene expression in the absence of vernalization.

## 2 MATERIALS AND METHODS

### 2.1 Screening for polymorphisms within the genomic region of *LanFTc1* in diverse germplasm

Polymorphisms in the *LanFTc1* genomic region were explored in a panel of 48 narrow‐leafed lupin accessions (Table S1) comprising (a) the species reference genome cultivar, Tanijl (Hane et al., 2017); (b) 43 accessions, including 30 genetically diverse wild accessions representing the natural geographic range of the species throughout the Mediterranean Basin (Mousavi‐Derazmahalleh et al., 2017) and 13 fully domesticated or semidomesticated accessions from Australia and Europe, all for which short‐read sequencing data were generated to assemble the *LanFTc1* region; (c) Krasnolistny, the first cultivar described as carrying the *Jul* early flowering time locus (Mikołajczyk, 1966); and (d) three Polish cultivars (Kazan, Mirela, and Sur) with Krasnolistny in their pedigree (Kubok, 1988).

The genomic sequence encompassing roughly 7‐Kb upstream and 2‐Kb downstream of the *LanFTc1* coding region was extracted from the Tanjil narrow‐leafed lupin reference genome (Hane et al., 2017) plus the 43 accessions with short‐read sequencing data by aligning Illumina Paired End reads from each accession to the Tanjil reference using Bowtie2 v2.2.9 (‐‐sensitive; Langmead & Salzberg, 2012). Variants were called using samtools and bcftools (Li, 2011; Li et al., 2009), which were then filtered to remove artefactual sequence variant calls arising from misalignments close to large (>1,000 bp) INDELs and polymorphisms that were physically disrupted by others.

Both Tanjil and the P27255 wild‐type *LanFTc1* sequence (GenBank ID KT862491) served as references to genotype the 1,423‐bp INDEL polymorphism previously identified by Nelson et al. (2017) in the 5' regulatory region and to survey for other alternative INDEL variations in this same region. After discovering additional INDELs in the 7‐Kb sequence upstream of *LanFTc1*, PCR primers were designed in the immediately adjacent sequences to screen for presence/absence of these INDELs for those accessions (Krasnolistny, Kazan, Mirela, and Sur) for which no re‐sequencing data were obtained. A summary of the INDELs, the different PCR primers assay, and the conditions for PCR amplification are provided in Table S2. To confirm the size and boundary of the newly identified INDELs, as determined from alignment of the short‐read sequences to the P27255 and Tanjil references, the nucleotide sequence of PCR products was determined by Sanger sequencing.

Additional INDELs and single nucleotide polymorphisms (SNPs) were also assessed in the coding and remaining noncoding sequences within the extracted *LanFTc1* genomic region in the 44 wild and domestic accessions using Tanjil as the reference genome. The P27255 wild‐type *LanFTc1* sequence (GenBank ID KT862491), encompassing approximately 5‐Kb upstream and 800‐bp downstream of the *LanFTc1* coding region, was used as the reference to call variants within the 1,423‐bp sequence in the 5' regulatory region that is deleted in the Tanjil reference genome.

### 2.2 Measuring degree‐days to flowering and vernalization responsiveness in diverse germplasm

The panel of 48 diverse accessions was phenotyped for time to flowering in two partially replicated trials (n = 1–3, as outlined in Table S1) for preliminary assessment of *LanFTc1* polymorphism genotype effect on vernalization responsiveness and time to flowering. The first trial included 40 accessions representing the 0‐, 1,208‐, and 1,423‐bp INDEL variants. Data were gathered for all accessions except for P22603, which failed to germinate. In the second trial, six accessions carrying the 5,162‐bp INDEL were compared with 10 representatives of the three other INDEL variants.

All seeds were germinated in Jiffy‐7^®^ peat pellets within a controlled environment room (CER) at The University of Western Australia (Perth, Australia), which was maintained at 20 °C constant temperature and with a 14‐hr photoperiod. Two vernalization treatments were provided: (a) a full vernalization treatment in which 2‐day old seedlings were transferred to a 4 °C room (14‐hr photoperiod) for 32 days before transferring to the CER for a further 140 days and (b) a mild, partial vernalization treatment, whereby 7‐day old seedlings were transferred to the 4 °C room for a total of 8 days before transferring to the CER for a further 140 days. A mild vernalization treatment was preferred to a fully non‐vernalizing treatment where accessions with a strong vernalization requirement would not flower at all. Thus, the mild vernalization treatment was designed to allow the degree of vernalization responsiveness to be measureable in the most strongly vernalization responsive accessions. All plants were transferred to the CER on the same day with approximately the same accumulated degree‐days (approximately 190 degree‐days, with 0 °C as the baseline temperature) and were placed in a randomized block design.

Flowering was scored immediately after anthesis, which was indicated by an erect standard petal (i.e., open flower) or the changing colour of petals. To accommodate the few accessions that did not flower within the allocated time of the experiment, even with mild vernalization treatment, flowering time was transformed to rate of flowering by taking the reciprocal of the degree‐days to flowering. Three‐way analysis of variance (ANOVA) was performed on the rate to flowering data using Genstat V.18, with vernalization treatment and deletion category as main effects, and accessions nested within deletion category to subdivide variance among and within categories. Category effects were compared using orthogonal contrasts by least significant difference. ANOVA was performed separately for each phenotyping trial, and residual plots used to identify outliers and check that errors were randomly and independently distributed.

### 2.3 Assessing the relationship of INDEL variation in the 5' regulatory region and *LanFTc1* gene expression

Following the preliminary analysis of *LanFTc1* polymorphism effect on phenotype, a subset of accessions representing four variants of prominent INDELs in the 5' regulatory region of *LanFTc1* was selected from the original panel of 48 accessions to phenotype more precisely flowering time and vernalization responsiveness and to correlate these traits with *LanFTc1* gene expression. The representative subset included the following narrow‐leafed lupins: (a) P27255, a wild Moroccan accession that is highly responsive to vernalization; (b) 83A:476, a vernalization‐insensitive Australian breeding line; (c) P22660, a wild Israeli accession with mild sensitivity to vernalization; (d) P29039, a vernalization‐insensitive Belarussian breeding line; and (e) Russian cultivar, Krasnolistny, also insensitive to vernalization. P27255 and 83A:476 are the parents for a wild × domestic F_8_ recombinant inbred line mapping population (Boersma et al., 2005; Kroc, Koczyk, Święcicki, Kilian, & Nelson, 2014; Nelson et al., 2006; Nelson, Moolhuijzen, et al., 2010).

Seeds were scarified and imbibed in Milli‐Q water for 6 hr before being immediately sown (non‐vernalized treatment) or incubated in a darkened room at 4 °C for 21 days in petri dishes (vernalized treatment). On the day of sowing the vernalized seeds, both treatments had accumulated approximately equal degree‐days (calculated using baseline temperature of 0 °C). All plants were grown in a phytotron located at The University of Western Australia (Perth, Australia) with a diurnal temperature range of 18 ± 0.5 °C (day) to 14 ± 0.5 °C (night) and exposed to natural photoperiod (10‐ to 12‐hr daylight during May to October 2017). Flowering time and degree‐days to flowering were scored immediately after anthesis and the data analysed as outlined above.

The four uppermost fully emerged leaves were harvested for gene expression analyses from three biological replicates per treatment per accession at five growth stages: 4‐leaf, 8‐leaf, 12‐leaf, 16‐leaf, and flowering. Samples were harvested between 12:00 and 14:00 hr and immediately snap‐frozen in liquid nitrogen. RNA isolation, cDNA synthesis, and quantitative reverse transcription PCR were conducted according to the methods of Taylor, Jost, Erskine, and Nelson (2016) and Nelson et al. (2017). Briefly, the relative expression of *LanFTc1* was calculated as the average cycle threshold (C_T_) for two primer pairs, which had previously been designed by Nelson et al. (2017) using transcript sequences from the draft Tanjil reference genome assembly (Kamphuis et al., 2015) to be specific to *LanFTc1* and to target unique portions of the coding sequence for gene‐wide transcription assessment. The average *LanFTc1* C_T_ value was then normalized against *Ubiquitin C*, which had been validated as a robust reference gene under the same experimental conditions (Taylor et al., 2016), and relative expression was finally expressed as 40‐ΔC_T_. This method reports relative transcript abundance on a Log_2_ scale, where a value of 40 represents the mean level of expression of *Ubiquitin C* and the fold difference between treatments is calculated as 2^ΔΔCT^ (where ΔΔC_T_ is equal to difference in average ΔC_T_ between vernalized and non‐vernalized treatments) when primer efficiency is approximately 2.0 (Bari, Pant, Stint, & Scheible, 2006).

Nested ANOVA and polynomial linear regression of relative gene expression over degree‐days to flowering were performed in Genstat V.18 as described above. Nested polynomial linear regression demonstrated nonsignificant differences between accessions within INDEL size categories, and therefore, only the INDEL size category results are presented (Figure 3a,b). The regressions were compared using orthogonal contrasts. The regression equations generated by Genstat were then used to plot smooth fitted curves, and we included the biological replicate data points for context (Figure 3a,b).

### 2.4 Characterizing the *LanFTc1* promoter region

Relative to P27255, representing the wild‐type *LanFTc1* sequence, three large, distinct INDELs were identified in the 5' regulatory region. Two of the INDELs, which were associated with modified gene expression and phenology, were assessed relative to the wild‐type for the presence/absence of transcription factor binding site motifs previously identified by Nelson et al. (2017). Putative binding site motifs were identified in that study using two open‐access web‐interface platforms, including JASPAR 2014, which contains CORE Plantae matrix models (Mathelier et al., 2014), and PLACE, which contains *cis*‐acting regulatory DNA elements in plants (Higo, Ugawa, Iwamoto, & Korenaga, 1999).

### 2.5 Assessment of linkage disequilibrium in the *LanFTc1* genomic region

To determine the likelihood of other polymorphisms within the *LanFTc1* genomic region being involved in modifying the response to vernalization, we measured the association of each polymorphism with vernalization responsiveness among the 44 accessions previously genotyped for polymorphisms in the *LanFTc1* genomic region. This was done by measuring pairwise linkage disequilibrium (*r* ^2^) of SNP and INDEL variants identified relative to the P27255 wild‐type *LanFTc1* reference sequence (see above) with the vernalization responsiveness phenotype, which was scored as a multi‐allelic genotype (unresponsive, mildly responsive, and responsive). An *r* ^2^ value was also calculated for the four INDEL variants as a single multi‐allelic polymorphism (0, 1,208, 1,423, and 5,162 bp). All pairwise *r* ^2^ values (‐‐ld‐window‐r2 0) were calculated using PLINK v1.9 (Purcell et al., 2007). Default filtering settings in PLINK were used to remove markers with low quality or that were almost monomorphic from analysis. A linear adjusted association analysis was also conducted in PLINK v1.9 to determine the significance and strength of the association between the sequence variants and vernalization responsiveness phenotype.

## 3 RESULTS

### 3.1 Screening for polymorphisms within the genomic region of *LanFTc1* in diverse germplasm

A 1,423‐bp deletion between 4,248‐ and 2,826‐bp upstream of the ATG start codon of *LanFTc1* was previously hypothesized as the causal sequence variant modifying vernalization responsiveness in the breeding line, 83A:476 (*Ku*), compared with the wild‐type *LanFTc1* sequence represented by P27255, and no polymorphisms were observed between accessions in the coding sequence (Nelson et al., 2017). To dissect this further, we surveyed the *LanFTc1* genomic region, from approximately 7‐Kb upstream to 2‐Kb downstream of the coding region in the Tanjil reference genome (Hane et al., 2017), for polymorphisms in 44 accessions of narrow‐leafed lupin. This analysis revealed a total of 260 SNPs and 56 INDELs relative to Tanjil, excluding the 1,423‐bp INDEL (Tables 1 and S3a). Importantly, no polymorphisms were found in the coding sequence. The genomic region features containing the most SNPs and INDELs (all less than 30 bp in length) were the large third intron and the 5' regulatory region, which contained approximately 50% and 32% of all polymorphisms. Variant calling using the P27255 wild‐type *LanFTc1* sequence revealed a further 17 SNPs and eight small INDELs (less than 20 bp in length) within the 1,423‐bp sequence in the 5' regulatory region, which is deleted in the Tanjil reference genome (Table S3b).

**Table 1:** A summary of SNP and INDEL polymorphisms (excluding the large INDEL within the 5' regulatory region) observed in the genomic region from approximately 7‐Kb upstream to 2‐Kb downstream of *LanFTc1* in 44 accessions of narrow‐leafed lupin relative to the Tanjil narrow‐leafed lupin reference genome

*LanFTc1* genomic region feature	Coordinates on pseudochromosome NLL‐10	Coordinates on scaffold_276_44	Number of SNP polymorphisms	Number of INDEL polymorphisms
5' regulatory region	8,016,843–8,023,566	3,823–10,546	91	11
5' UTR	8,023,567–8,023,842	10,547–10,822	1	1
Exon 1 (CDS)	8,023,843–8,024,040	10,823–11,020	0	0
Intron 1	8,024,041–8,024,162	11,021–11,142	1	0
Exon 2 (CDS)	8,024,163–8,024,225	11,143–11,205	0	0
Intron 2	8,024,226–8,024,381	11,206–11,361	2	3
Exon 3 (CDS)	8,024,382–8,024,420	11,362–11,400	0	0
Intron 3	8,024,421–8,030,939	11,401–17,919	127	29
Exon 4 (CDS)	8,030,940–8,031,155	17,920–18,135	0	0
3' UTR	8,031,156–8,031,424	18,136–18,404	3	2
3' regulatory region	8,031,425–8,033,155	18,405–20,135	35	10
Total			260	56

*Note*. Genomic region features include regulatory regions adjacent to the coding sequence (CDS), and the untranslated regions (UTRs), exons (coding sequences), and introns (noncoding intragenic sequences) of *LanFTc1*. SNP = single nucleotide polymorphism; INDEL = insertion/deletion.

**Figure 1:**
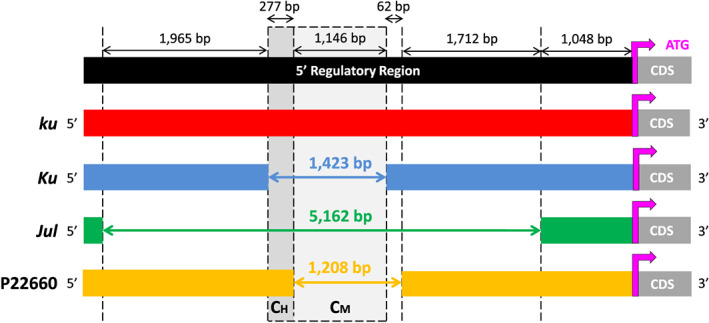
A schematic illustrating the positions of insertion/deletion genotypes in the 5' regulatory region of *LanFTc1* relative to the start codon (ATG) of the coding sequence (CDS). The wild‐type *LanFTc1* sequence (*ku*) was obtained from P27255, a wild Moroccan accession, and the 1,423‐bp deletion (*Ku*) from the Tanjil reference genome. A 5,162‐bp deletion (*Julius* or *Jul*) was found in several European breeding lines and cultivars, including Krasnolistny (Russian cultivar), P29039 (Belarussian breeding line), and Emir (Polish cultivar). A 1,208‐bp deletion was identified in P22660, a wild accession from Israel. Critical regions of the regulatory region, if deleted, enable high (C_H_, shaded dark grey) and moderately high (C_M_, shaded light grey) levels of *LanFTc1* expression, respectively, relative to the wild‐type sequence

We then used P27255 and Tanjil as references to genotype the 1,423‐bp INDEL and determine if there were any other major INDEL variations in the 5' regulatory region in 42 additional wild and domestic accessions. The wild‐type *LanFTc1* sequence was present in a total of 29 wild accessions from the Mediterranean and two vernalization responsive Australian cultivars (all known to carry the *ku* allele), whereas the 1,423‐bp deletion was present in 10 Australian and Polish *Ku* cultivars. Importantly, two new large INDEL variants were identified. The first of these was a 1,208‐bp deletion observed between 3,970‐ and 2,763‐bp upstream of the ATG start codon in the Israeli accession, P22660, relative to the P27255 wild‐type *LanFTc1* sequence. The majority of this smaller deletion overlapped with that of the 1,423‐bp variant. However, as determined from Sanger sequencing (GenBank ID MH166758), the first 277 bp at the 5' end of the 1,423‐bp deletion was retained in P22660, whereas a further 62 bp immediately downstream of the 1,423‐bp variant had been deleted (Figure 1). The second INDEL variant was a prominent deletion of 5,162 bp in P29039 (a Belarussian breeding line) and Emir (a Polish cultivar) positioned between 6,209‐ and 1,048‐bp upstream of the ATG start codon relative to the P27255 reference. Sanger sequencing supported a shared origin of the 5,162‐bp deletion as both P29039 and Emir had identical deletion breakpoints (GenBank ID MH166759). This large deletion completely spanned the 1,423‐ and 1,208‐bp deletions (Figure 1).

To determine if the narrow‐leafed lupin cultivars carrying *Jul* contain the wild‐type sequence or one of the three deletion genotypes, a PCR‐marker (Table S2) approach was used. This confirmed that Krasnolistny, the original *Jul* cultivar, and three Polish cultivars known to descend from it (Kazan, Mirela, and Sur) contain the 5,162‐bp deletion as detected in P29039 and Emir (Figure S1).

### 3.2 Measuring degree‐days to flowering and vernalization responsiveness in diverse germplasm

**Figure 2:**
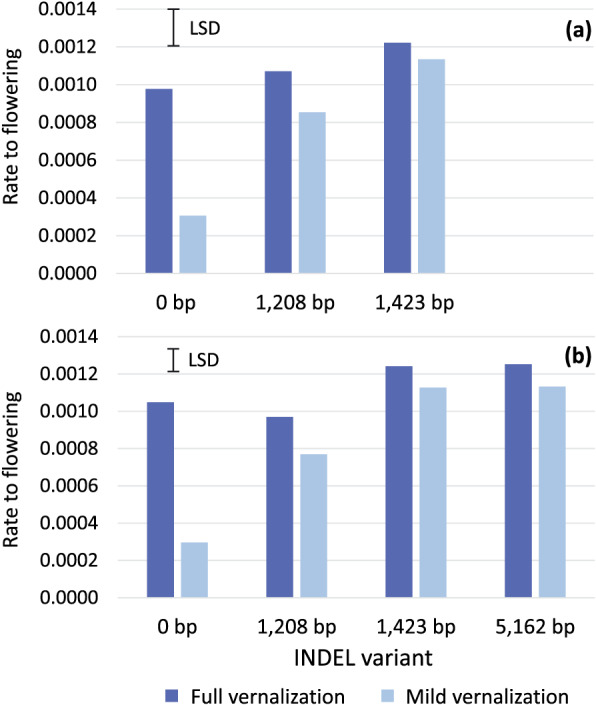
Average rate to flowering (reciprocal of degree‐days to flowering) in mildly and fully vernalizing conditions for narrow‐leafed lupins possessing various insertion/deletions (INDELs) in the 5' regulatory region of *LanFTc1* in two phenotyping trials. (a) Trial 1 included narrow‐leafed lupins carrying the 0‐bp deletion (*n* = 29), 1,208‐bp deletion (*n* = 1), and 1,423‐bp deletion (*n* = 9). (b) Trial 2 included narrow‐leafed lupins with the 0‐bp deletion (*n* = 2), 1,208‐bp deletion (*n* = 1), 1,423‐bp deletion (*n* = 8), and 5,162‐bp deletion (*n* = 6). The least significant difference (LSD) value is provided to compare responses within and between vernalization treatments in each phenotyping trial ([a] LSD = .00020; [b] LSD = .00011)

To explore whether the four prominent INDEL variants in the 5' regulatory region of *LanFTc1* may affect vernalization responsiveness and phenology in narrow‐leafed lupin, we phenotyped rate to flowering (reciprocal of degree‐days to flowering) in the full panel of 48 accessions under both mildly and fully vernalizing conditions across two trials, with 39 accessions in the first trial (Figure 2a) and 17 accessions in the second trial (Figure 2b; Table S1). In both trials, there were strong INDEL variant by vernalization treatment interactions (*p* < .001), in which vernalization response was consistently proportional to flowering time (Figure 2a,b). Thus, the strongest vernalization response was observed in the late flowering wild‐type accessions (0‐bp INDEL), followed by the intermediate flowering 1,208‐bp INDEL accession and finally, the early flowering 1,423‐ and 5,162‐bp accessions, both of which had a small response to vernalization (Figure 2b). Accordingly, the large differences in rate to flowering observed between the 0‐, 1,208‐, 1,423‐ and 5,162‐bp variants under mild vernalization were greatly reduced under full vernalization. Thus, under fully vernalizing conditions, the strongly vernalization responsive wild‐types (0‐bp deletions) flowered at the same rate as the intermediate 1,208‐bp INDEL variant and only marginally slower than the weakly vernalization responsive accessions with the 1,423‐ and 5,162‐bp deletions.

### 3.3 Assessing the relationship of INDEL variation in the 5' regulatory region upon *LanFTc1* gene expression

On the basis of the association between phenology and vernalization responsiveness with INDEL variation in the 5' regulatory region of *LanFTc1*, we measured *LanFTc1* gene expression in five representative accessions, grown with and without vernalization treatment. Four of the accessions included P27255, P22660, 83A:476, and P29039, representing the 0‐bp (*ku*), 1,208‐bp, 1,423‐bp (*Ku*), and 5,162‐bp deletions, respectively. Krasnolistny was also included to gain further evidence implicating the 5,162‐bp deletion in the 5' regulatory region of *LanFTc1* as the causal mutation for the *Jul* locus.

#### 3.3.1 Flowering time is earlier, and vernalization responsiveness is reduced or effectively lost in accessions with a deletion genotype

Orthogonal contrasts revealed that the flowering behaviour of the representative subset was consistent with the larger association study (Figure 2). INDEL genotype by vernalization treatment interactions was highly significant (*p* < .001) and ranked in the same order as previously. Thus, the wild‐type 0‐bp deletion was much more vernalization responsive than the 1,208‐bp deletion (*p*_diff_ < .001), which in turn was more responsive than the 1,423‐ and 5,162‐bp INDELs (*p*_diff_ < .001), which had a similar low response to vernalization (*p*_diff_ = .883; Table 2). The two accessions with the 5,162‐bp deletion genotype also had a similar low response to vernalization (*p*_diff_ = .450), although P29039 was always slightly later flowering than Krasnolistny (*p*_diff_ < .001; Table 2).

**Table 2:** Average days and degree‐days to flowering in vernalized and non‐vernalized treatments for narrow‐leafed lupins representing the 5' regulatory region wild‐type sequence (*ku*) for *LanFTc1* and three major deletion variants of 1,208 bp, 1,423 bp (*Ku*), and 5,162 bp (*Jul*)

		Days to flowering		Degree‐days to flowering	
Accession	Deletion genotype	Vernalized	Non‐vernalized	Vernalized	Non‐vernalized
P27255	0 bp (*ku*)	53.7	134.3	1,023.2	2,434.8
P22660	1,208	49.0	67.0	941.5	1,256.5
83A:476	1,423 bp (*Ku*)	51.0	49.0	976.5	941.5
P29039	5,162 (*Jul*)	58.3	55.7	1,104.8	1,058.2
Krasnolistny	5,162 (*Jul*)	52.3	50.7	999.8	970.7

*Note*. Differences between accession means within and across treatments greater than 1.9 days and 33.64 degree‐days are significant (least significant difference *p* < .05).

#### 3.3.2 *LanFTc1* is expressed to varying degrees in accessions with a deletion genotype independently of vernalization treatment

**Figure 3:**
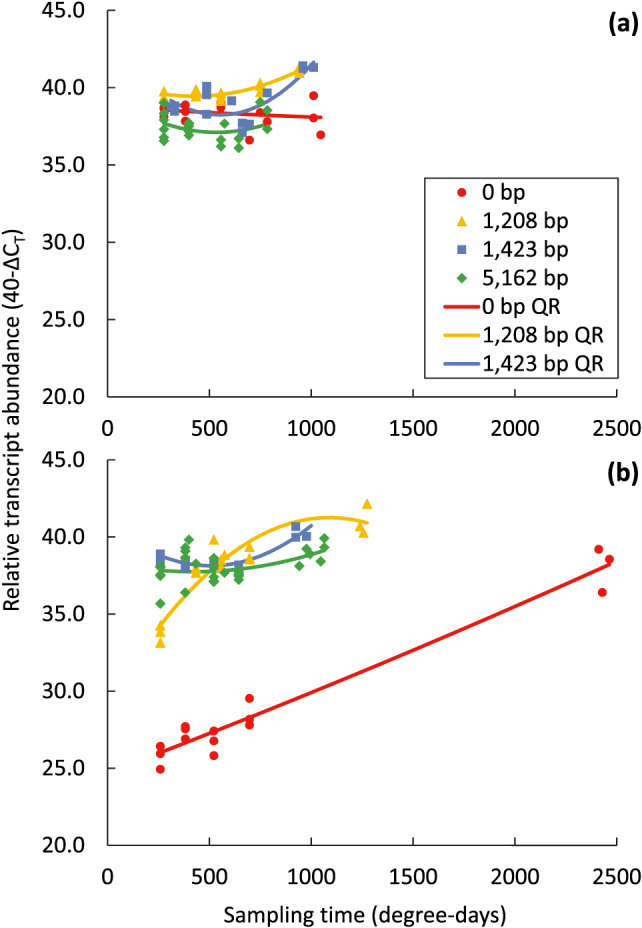
Relative expression of *LanFTc1* at various degree‐days from 4‐leaf stage (approximately 277 degree‐days) to first flowering in deletion categories of narrow‐leafed lupin (red circle, 0‐bp deletion wild‐type; yellow triangle, 1,208‐bp deletion; blue square, 1,423‐bp deletion; and green diamond, 5,162‐bp deletion) with (a) and without (b) vernalization. The quadratic regression (QR) model captured 94.7% of variance and indicated significant intercept, linear, and quadratic slope differences (*p* < .001) between category/vernalization treatment combinations. The final sampling time is at first flower and therefore varies widely between treatment combinations

All accessions had high gene expression under vernalizing conditions during vegetative growth, starting from a common high basal level at the 4‐leaf stage (approximately 277 degree‐days, Figure 3a). The deletion size categories (1,208, 1,423, and 5,162 bp) had similar curvilinear increases in relative transcript abundance in the late vegetative stage (approximately 750 degree‐days), whereas the wild‐type (0‐bp deletion) showed a flat slope, with no change in transcript abundance over time (*p* = .851).

Relative *LanFTc1* expression varied greatly among the accessions in the absence of vernalization (Figure 3b). Those with the 1,423‐ and 5,162‐bp deletions behaved similarly to their respective vernalized treatments, with similar curvilinear increases in transcript abundance in the late vegetative stage. By contrast, relative expression levels in the accession (P22660) with the smaller 1,208‐bp deletion rose rapidly from an intermediate basal level and reached similar levels as the larger deletion categories by the mid‐vegetative stage (Figure 3b). Finally, the wild‐type (0‐bp deletion) had a slow linear increase in relative gene expression throughout the vegetative phase, starting from the lowest level of expression at the 4‐leaf stage, and reached similar levels to the three deletion categories by the onset of flowering (Figure 3b).

### 3.4 Characterizing the *LanFTc1* promoter region

The gene expression profiles of the three deletion variants indicate that sections of the 5' regulatory region are critical for regulating flowering time via the vernalization pathway and *LanFTc1*. It appears that the 1,423‐ and 5,162‐bp deletions are functionally equivalent, as they both result in insensitivity to vernalization and similar *LanFTc1* expression profiles (Figure 3a,b). Therefore, crucial regulatory elements responsible for full gene suppression in the absence of vernalizing conditions should reside within the 1,423‐bp INDEL sequence (Figure 1). The functional activity of the 1,208‐bp INDEL further refines this critical region. As the first 277 bp at the 5' end of the 1,423‐bp INDEL is not also deleted within the 1,208‐bp INDEL, it suggests that this 277‐bp region is critical for establishing complete derepression of *LanFTc1* (C_H_; critical region for high early expression and vernalization insensitivity). Additionally, as the 62‐bp sequence at the 3' end of the 1,208‐bp deletion is conserved in the wild‐type, this indicates that the 1,146‐bp sequence common to the 1,208‐, 1,423‐, and 5,162‐bp INDELs is responsible for establishing a moderate level of early gene activity without vernalization (C_M_; critical region for moderate early expression and moderate vernalization responsiveness).

We next explored variation in the C_H_ and C_M_ critical regions that may explain differences in vernalization responsiveness and *LanFTc1* expression. Both critical regions were screened for candidate transcription factor binding motifs that may have roles in the repression of *LanFTc1* within the P27255 representative wild‐type sequence from the comprehensive list compiled by Nelson et al. (2017). A total of 31 individual motifs were found within the C_H_ region, including six in which the motif overhangs the C_H_ region and the adjacent 5' wild‐type sequence and/or contains an alternative SNP allele in one or more vernalization responsive accession(s) (Tables S4 and S5). Meanwhile, as many as 168 individual motifs were identified within the C_M_ region, including seven for which one or more vernalization responsive accession(s) have SNPs (Tables S4 and S5). Among all of the motifs identified upstream of the coding sequence within the 5' regulatory region and 5' untranslated region (UTR), the binding sites for three and five types of transcription factors were unique to the C_H_ and C_M_ regions, respectively, including several reported to have roles in determining flowering time (Table 3). Only a single type of transcription factor, named BRI1‐EMS‐SUPPRESSOR 1 (BES1), was common to both critical regions yet absent in the remainder of the adjacent 5' UTR and 5' regulatory region.

**Table 3:** A list of candidate transcription factor (TF) binding site motifs unique to sequences within the 5' regulatory regions critical for establishing moderate (C_M_) or high (C_H_) levels of derepressed *LanFTc1* expression during early vegetative growth

Motifs present in the wild‐type sequence	Motifs present in the C_H_ sequence	Motifs present in the C_M_ sequence	Role of TF in flowering within other angiosperms	References
AGL9	1^a^	0	AGAMOUS‐LIKE 9 (AGL9), also known as SEPALLATA3 (SEP3), is a MADS‐box TF that is involved in establishing identity of petals, stamens, and carpels in *Arabidopsis*. In rice (*Oryza sativa*), knock out of two *AGL9/SEP3* homologues, *OsMADS7* and *OsMADS8*, also results in delayed flowering.	Mandel and Yanofsky (1998) Pelaz, Ditta, Baumann, Wisman, and Yanofsky (2000) Cui et al. (2009)
ATHB5	1	0	ARABIDOPSIS THALIANA HOMEOBOX PROTEIN 5 (ATHB5) is a homeodomain leucine zipper TF that forms a heterodimer with its family member, ARABIDOPSIS THALIANA HOMEOBOX PROTEIN 16, which regulates photoperiodic responsiveness in *Arabidopsis*.	Johannesson, Wang, and Engström (2001) Y. Wang et al. (2003) De Smet et al. (2013)
PI	1	0	PISTILLATA (PI) is a MADS‐box TF that is involved in establishing identity of petals and stamens in *Arabidopsis*.	Hill and Lord (1989) Bowman, Smyth, and Meyerowitz (1989)
AT3G20750	0	1	AT3G20750 (also known as GATA29) is a member of the GATA protein family and contains a HAN domain, which has roles in regulating cell differentiation and speciation, including for floral organs, in *Arabidopsis*. The rice (*Oryza sativa*) *AT3G20750* homologue, *NECK LEAF 1* (*NL1*), has a similar role in floral organ identity, and its overexpression is thought to affect regulation of *Hd3a*, a rice *FT* homologue, and delay flowering.	Reyes, Muro‐Pastor, and Florencio (2004) Zhao et al. (2004) Behringer and Schwechheimer (2015) L. Wang et al. (2009x) Tamaki, Matsuo, Wong, Yokoi, and Shimamoto (2007)
C1	0	1	C1 is a MYB TF involved in anthocyanin biosynthesis, thus flower colouration, in maize (*Zea mays*).	Paz‐Ares, Ghosal, Wienand, Peterson, and Saedler (1987) Sainz, Grotewold, and Chandler (1997) Mola, Grotewold, and Koesa (1998)
MADSA	0	1	MADSA, also known as AGAMOUS‐LIKE 20 and SUPPRESSOR OF OVEREXPRESSION OF CONSTANS 1 (SOC1) in *Arabidopsis*, is a MADS‐box TF activated in the shoot apical meristems during the transition from vegetative to floral development, and which integrates signals from the giberellin pathway, and also the photoperiod pathway through CONSTANS via *FT*.	Borner et al. (2000) Yoo et al. (2005)
NAC6	0	1	NAC6 (also known as NAC2 or AtNAC2) is a member of the NAC TF family, which has roles in regulating morphogenesis and stress responses, and is itself involved in the regulation of stamen development in *Arabidopsis*.	Ooka et al. (2003) Mandaokar et al. (2006)
WRKY	0	1	WRKY20 is a member of the WRKY TF family that has roles in plant stress responses and development. Wild soybean (*Glycine soja*) homologue *WRKY20* is abundantly expressed in flowers and floral meristems and is thought to be involved in positive regulation of the autonomous pathway. Its overexpression in *Arabidopsis* results in early flowering and is associated with up‐regulation of floral integrator genes, including *FT* and *SOC1*.	Luo et al. (2013)
BES1	1	2	BES1 is a member of the BES1/BZR1 TF family and interacts with EARLY FLOWERING 6 and RELATIVE OF EARLY FLOWERING 6 in *Arabidopsis* to repress the photoperiodic pathway and *FLOWERING LOCUS C*, a repressor of *FT*.	Noh et al. (2004) Yu et al. (2008)

a Note that six of seven nucleotides forming this motif are located within the C_H_ region and that one or more vernalization responsive accessions contain a single nucleotide polymorphism within the motif.

### 3.5 Assessment of linkage disequilibrium in the *LanFTc1* genomic region

**Figure 4:**
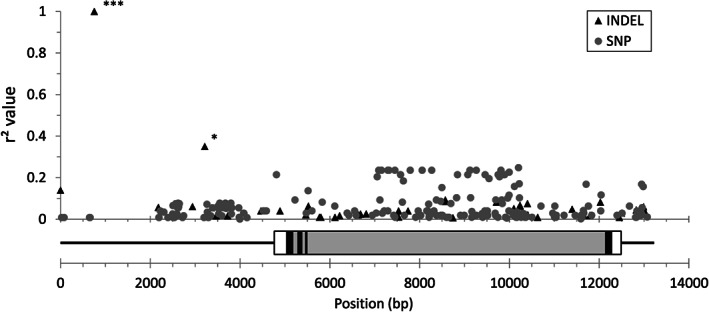
Linkage (represented as *r* ^2^) of insertion/deletions (INDELs; black triangles) and single nucleotide polymorphisms (SNPs; grey circles) identified in the narrow‐leafed lupin *LanFTc1* wild‐type genomic sequence, represented by Moroccan accession P27255, with vernalization responsiveness. The positions of polymorphisms are indicated relative to the base pair (bp) position along the wild‐type *LanFTc1* genomic sequence (GenBank ID KT862491) and a schematic of the *LanFTc1* genomic features, including regulatory regions (solid black line), untranslated regions (solid white bar with black border), exons (solid black bar with black border), and introns (solid grey bar with black border). An *r* ^2^ value of 1.0 represents perfect linkage with vernalization response phenotype. Asterisks denote significant associations between polymorphisms and the vernalization responsiveness phenotype (Bonferroni adjusted *p* values: *.01 < *p* < .05; **.001 < *p* < .01; ***p < .001)

To rule out the involvement of other polymorphisms in the *LanFTc1* gene region being involved in modifying vernalization responsiveness, we measured the association of each polymorphism with the vernalization responsiveness phenotype. A total of 48 INDELs and 206 SNPs were used for pairwise calculation of linkage disequilibrium (*r* ^2^) with the vernalization responsiveness genotype. Low *r* ^2^ values of .35 or less were observed for the vast majority of polymorphisms (Figure 4). One INDEL point at 752 bp in the P27255 wild‐type reference sequence, which represents the 1,423‐bp deletion in the 5' regulatory region, was the only variant with an *r* ^2^ value of 1.0 and perfect linkage with the vernalization responsiveness phenotype (Figure 4). An *r* ^2^ value of 1.0 was also achieved when grouping the 1,423‐, 5,162‐, and 1,208‐bp deletions together as a single multi‐allelic INDEL. The major association between the 1,423‐bp INDEL and vernalization responsiveness phenotype was highly significant (PLINK linear regression, coefficient *t* statistic = 32.58, Bonferroni adjusted *p* = 6.60e^−27^). There was an additional small association with vernalization responsiveness for a 1‐bp INDEL at position 3,215 bp relative to the P27255 wild‐type sequence (PLINK linear regression, Bonferroni adjusted *p* = .04; Figure 4).

## 4 DISCUSSION

### 4.1 A series of *cis*‐regulatory variants in a legume *FT* homologue

A 1,423‐bp deletion in the 5' regulatory region of *LanFTc1*, an *FT* homologue, was recently hypothesized as the causal mutation behind the *Ku* locus that has been significant in establishing narrow‐leafed lupin as a viable pulse crop in Australia and northern Europe (Nelson et al., 2017). Here, we have shown that another two independently occurring mutations, namely, 1,208‐ and 5,162‐bp deletions, overlap the same region of the promoter, creating a series of *cis*‐regulatory variants that derepress *LanFTc1* expression to varying extents. Additionally, we have shown that the 1,423‐bp deletion, plus all three deletions when scored as a singular multi‐allelic variant at the same position, are the only sequence variants in the *LanFTc1* wild‐type sequence that are perfectly associated with vernalization response and, as a consequence, early flowering time under non‐vernalizing conditions. The remaining 206 SNPs and 48 small INDELs within the 13‐Kb wild‐type genomic region are not associated with vernalization responsiveness (*r* ^2^ ≤ .35; Figure 4), with the exception of one small INDEL (1 bp), which is weakly linked (*r* ^2^ = .35; Figure 4). This finding compliments those of Nelson et al. (2017), where presence/absence of the 1,423‐bp deletion was perfectly predictive of vernalization responsiveness in 216 accessions and provides compelling evidence that the INDEL series are the likely causal mutations modifying vernalization responsiveness and, thus, phenology. However, we have not yet ruled out the possibility that other variants outside of this 13‐Kb genomic region are also strongly or perfectly associated with vernalization responsiveness. Lastly, we found strong evidence that the 5,162‐bp deletion corresponds to *Jul*, supporting previous speculation that these two dominant early flowering time genes discovered independently in Australian and European breeding programs (Rahman & Gladstones, 1972) are in fact different alleles of the same gene, *LanFTc1*. We are currently developing biparental genetic populations to confirm this conclusion.

To the best of our knowledge, this is the first report of a naturally occurring series of mutations in the noncoding region of a floral integrator gene in any legume species. However, it adds to a growing list of literature similarly reporting series of *cis*‐regulatory variants of vernalization and photoperiodic pathway genes in *Arabidopsis* and cereal crops. The largest and most widely published allelic series identified to date involves *VRN‐1*, a MADS‐box transcription factor that is orthologous to *APETALA1* in *Arabidopsis* (Yan et al., 2003) and that is involved in maintaining down‐regulation of floral repressors following vernalization within members of the Poaceae family (A. Chen & Dubcovsky, 2012). Within the promoter and intronic regions, a staggering number of INDELs ranging in size from 20 to 6,850 bp, in addition to a single SNP, have been identified in the A, B, D, and G genomes of various diploid, tetraploid, and hexaploid wild and domestic wheats and their progenitors (Fu et al., 2005; Golovnina, Kondratenko, Blinov, & Goncharov, 2010; Konopatskaia, Vavilova, Kondratenko, Blinov, & Goncharov, 2016; Milec, Tomková, Sumíková, & Pánková, 2012; Muterko, Balashova, Cockram, Kalendar, & Sivolap, 2015; Santra, Santra, Allan, Campbell, & Kidwell, 2009; Shcherban, Efremova, & Salina, 2012; Takumi, Koyamam, Fujiwara, & Kobayashi, 2011; Yan et al., 2004; Zhang, Gao, Wang, Chen, & Cui, 2015), plus the H genome of barley (*Hordeum vulgare* L.; Fu et al., 2005). Our study also adds to others in *Arabidopsis* (Liu et al., 2014; Schwartz et al., 2009), perennial ryegrass (*Lolium perenne*; Skøt et al., 2011), and wheats and barley (F. Chen et al., 2013; Yan et al., 2006) showing that *FT* orthologues have similarly been a common target for the evolution of natural flowering time variation in a range of plant families. Lastly, a series of 7‐bp tandem repeat INDELs has also been identified in *Arabidopsis* to modify the *cis*‐regulation of *CONSTANS* (*CO*), a gene which encodes a zinc‐finger transcription factor responsive to the photoperiodic and circadian clock flowering pathways (Rosas et al., 2014).

### 4.2 Implications for breeding and expansion of the adaptive range of narrow‐leafed lupin

Discovery of an INDEL series in the promoter region of *LanFTc1* has major practical implications in light of its demonstrated capacity to modify vernalization responsiveness and flowering time in narrow‐leafed lupin. The *Ku* (1,423‐bp deletion) and *Jul* (5,162‐bp deletion) alleles have already been widely incorporated into domestic breeding programs in Australia and Europe. However, the 1,208‐bp deletion present in the Israeli wild accession, P22660, represents a new form of valuable variation that has the potential to delay flowering time by approximately 2.5 weeks in the absence of vernalization. Such variation would be extremely beneficial in expanding the production range of narrow‐leafed lupin, plus increasing crop adaptation and yield potential in current environments with longer seasons, such as the southern Western Australian and eastern Australian growing regions. The predominance of the *Ku* and *Jul* alleles in breeding programs means that, without prior knowledge of the 1,208‐bp INDEL variant, it would not be easily identified in the early stages of segregation from hybrids with breeding lines containing the dominant early alleles, *Ku* or *Jul*. The PCR marker designed by Nelson et al. (2017) will serve as a useful resource to screen for the 1,208‐bp INDEL in future breeding (Table S2a).

Similar to the 1,208‐bp deletion identified in wild germplasm from Israel, it is interesting to note that *Jul* is thought to have originated from the same region of the Middle East (Mikołajczyk, 1966). Evaluation studies of previous germplasm collection trips (Clements & Cowling, 1994; Gladstones & Crosbie, 1979) and a recent genetic and adaptive diversity analysis (Mousavi‐Derazmahalleh et al., 2017) have identified the Eastern Mediterranean and Northern Africa (including but not limited to parts of Morocco, the Middle East, and Aegean islands) as key geographic regions associated with early phenology in the natural habitat of *L*. *angustifolius*. The lower elevation and latitude of these regions, in combination with reduced, variable rainfall and increased seasonal temperatures, results in shorter growing seasons with heightened abiotic stresses that drive phenological evolution (Berger, Ludwig, & Buirchell, 2008; Berger, Shrestha, & Ludwig, 2017). Therefore, it is possible that valuable *cis*‐regulatory variations of *LanFTc1* or other genes regulating time to flowering exist in wild populations of narrow‐leafed lupin from these origins. We are currently exploring this possibility in our research activities.

### 4.3 Understanding the regulation of *FT* homologues and the mediation of vernalization responsiveness in the legume family

In addition to the potential benefits to narrow‐leafed lupin breeding, the discovery of two new INDEL variants within the 5' regulatory region has also enabled us to explore which part of the promoter is critical for retaining normal repression of *LanFTc1* in the absence of vernalization and, which if manipulated, is capable of modifying phenology. This critical region has been further divided into two zones, one of which is critical for establishing a medium level of derepressed gene expression (C_M_), whereas the second enables high and completely derepressed transcriptional state (C_H_). However, at this stage, it remains unclear as to why these regions are critical and what role(s) the deleted sequences play in the wild‐type *ku* allele.

As evidenced from variant series in other species and flowering time genes, *cis*‐regulatory changes are mediated by polymorphisms in a number of different ways. The classical *FT* promoter in *Arabidopsis* contains four major blocks (Adrian et al., 2010; Liu et al., 2014), comprising first, the A block, positioned roughly 400‐bp upstream of the ATG start codon and which contains a number of transcriptional elements, such as those bound by CO (Tiwari et al., 2010); second, the B block, located approximately 1.8‐Kb upstream of the coding sequence and containing two conserved binding sequences for basic helix‐loop‐helix proteins (Adrian et al., 2010); the distal C block, located roughly 5.2‐Kb upstream of the start codon and which contains binding elements for proteins involved in delivering CO to motifs within Block A (Cao et al., 2014); and lastly, an intermittent sequence roughly 3.7‐Kb upstream of the ATG transcription start site that includes a block known as ID, which is involved in establishing physical proximity of the A and C blocks for photoperiod responsiveness. It is the latter in which two INDELs influencing promoter efficiency have evolved in *Arabidopsis* (Liu et al., 2014). The first of these includes an approximately 1.1‐Kb insertion near Block ID that causes an excessive physical distance between Blocks A and C, preventing their normal interaction. Meanwhile, a smaller deletion of approximately 250 bp contrastingly provides sufficient proximity of Blocks A and C, such that the ID block is redundant. Previous research from Książkiewicz et al. (2016) has indicated a lack of sequence conservation between the 1,423‐bp deletion (*Ku*) and ID block, therefore suggesting that the INDEL series in *LanFTc1* is unlikely to operate in a similar manner to that of the *FT* series in *Arabidopsis*. The discovery of the 5,162‐bp deletion in *Jul* accessions in this study also suggests that this is not the case, as a significantly large proportion of the promoter that may correspond to other blocks has been deleted. However, further research to characterize the sequences either side of the 5,162‐bp INDEL may be required to firmly eliminate the improvement of promoter efficiency by modification to regulatory element proximity as one possible consequence of the INDEL series in *LanFTc1*.

Alternative ways in which the INDEL series may instead modify *cis*‐regulation of *LanFTc1* is through changing the profile of transcription factor binding sites within the promoter region or their capacity to be bound. A straightforward explanation is the complete removal of transcription factor binding site motifs from within the three deletions. We refined a list of candidate transcription factor motifs from Nelson et al. (2017), revealing a total of 168 and 31 individual motifs present in the wild‐type promoter sequence yet which are absent in the C_M_ and C_H_ regions, respectively (Tables S4 and S5). However, this is still an extremely large number of candidate transcription factor motifs, and it will be very difficult to further resolve which may or may not have functional roles in *LanFTc1* regulation, especially if no further variants are found overlapping this region.

Copy number of transcription factor binding sites also represents another possibility of *cis*‐regulatory modification. As has been demonstrated in *Arabidopsis*, increasing from three to four tandem repeats of a 7‐bp motif for CYCLING DOF FACTOR 1 in the promoter of *CO* increases the day‐time repression of this gene and significantly delays flowering time (Rosas et al., 2014). We identified motifs for the binding site of a single transcription factor, named BES1, present once within the C_H_ and twice within the C_M_ critical regions (Tables 2, S4, and S5), yet nowhere else in the 5' UTR and 5' regulatory region of *LanFTc1*. In *Arabidopsis*, BES1 interacts with EARLY FLOWERING 6 and RELATIVE OF EARLY FLOWERING 6 proteins to respectively repress the photoperiodic pathway, through unknown means, and *FLOWERING LOCUS C*, a repressor of *FT* that is itself repressed by vernalization (Noh et al., 2004; Yu et al., 2008). Therefore, although both *FLOWERING LOCUS C* and *RELATIVE OF EARLY FLOWERING 6* are apparently absent from the narrow‐leafed lupin genome (Hane et al., 2017), there is precedence for BES1 involvement in the regulation of flowering time, and it is conceivable that it could be involved in the direct regulation of *LanFTc1* through partnership with other flowering time genes. In such a scenario, deletion of two copies of the BES1 binding site motif via the 1,208‐bp INDEL genotype would be sufficient to elevate *LanFTc1* expression to an intermediate level, and deletion of all three motifs via the 1,423‐ or 5,162‐bp INDELs would fully derepress expression. With genome editing tools, such as the CRISPR/Cas‐9 system (Bortesi & Fischer, 2015), and more efficient transformation protocols in narrow‐leafed lupin (Barker et al., 2016), it may be feasible to modify BES1 binding site motifs in the wild‐type sequence to test the validity of this hypothesis in the future. If BES1 has a role in regulating *LanFTc1*, BES1's known involvement within the photoperiodic pathway could explain why cultivars with the 1,423‐bp deletion are also less responsive to inductive long days than wild‐types without the large deletion in the promoter region of *LanFTc1* (J. D. Berger, unpublished data).

Lastly, the location of INDELs relative to transcription factor binding site motifs can influence the affinity for transcription factor binding, as demonstrated in the case of the *FT* homologue allelic series in perennial ryegrass (Skøt et al., 2011). Relative to the wild‐type sequence designated as the C haplotype, a deletion of five nucleotides positioned 7‐ to 11‐bp downstream of a conserved motif (the A haplotype) resulted in a 2‐day delay in flowering time, whereas a six nucleotide deletion positioned 1 to 6 bp directly 3' of the conserved motif (the B haplotype) resulted in a 7‐day flowering time delay. Here, we have identified three classes of transcription factor binding site motifs, which are disrupted by the 5' end of the C_H_ region and which are completely absent in the C_M_ region. However, motifs for these same transcription factors are also found on several occasions elsewhere within the *LanFTc1* genomic region, and small INDEL polymorphisms present in vernalization responsive accessions without the 1,208‐, 1,423‐, or 5,162‐bp INDELs can also be found disrupting some of these motifs. Therefore, it seems unlikely that any of these motifs are functional or critical to *LanFTc1*; however, further research will be required to better characterize other motifs adjacent to the large deletions.

Despite our lack of knowledge as to how they impact gene expression, the discovery of the INDEL variant series in the 5' regulatory region of *LanFTc1* has provided us with a rare opportunity to better explore possible ways in which *FT* homologues are regulated, and vernalization responsiveness is mediated, at the molecular level outside of the Brassicaceae and Poaceae. At present, our greatest understanding concerning vernalization response within the legume family comes from *Medicago truncatula* (Weller & Ortega, 2015). In this model species, an *FTa1* homologue is up‐regulated following the return of warm conditions post‐vernalization, and loss‐of‐function mutations within the coding sequence render plants insensitive to vernalization (Laurie et al., 2011). Three induced mutant lines with dominant early, vernalization‐insensitive flowering have been shown to contain transposon insertions in the large third intron or the 3' regulatory region that result in up‐regulated expression of *FTa1*, suggesting that these genomic regions are important sites for conferring transcriptional repression in the wild‐type (Jaudal et al., 2013). However, similar to the present story in narrow‐leafed lupin, it is unknown what elements within these regions are important for the vernalization pathway and *FTa1* transcription. Thus far, it appears that methylation in the *FTa1* genomic region is unlikely to play a role, with no differences observed between the mutants with induced transposon insertions and wild‐type plants (Jaudal et al., 2013). The discovery of further INDEL variants in *LanFTc1* could provide further clues of how signalling mediated through the vernalization pathway is centred on *FT* homologues at the molecular level in the Fabaceae.

## Supporting information


**Figure S1.** PCR markers to assay four major INDEL variants (0 bp, 1,423 bp, 1,208 bp and 5,162 bp) in the promoter region of *LanFTc1,* a *FLOWERING LOCUS T* homologue of narrow‐leafed lupin.

**Table S1.** Narrow‐leafed lupin (*Lupinus angustifolius* L.) germplasm genotyped for polymorphisms in the genomic region (approximately 7 Kb upstream and 2 Kb downstream of the coding sequence) of *LanFTc1*, a vernalisation responsive *FT* homologue.
**Table S2.** a) Primers, b) PCR reaction, and c) cycling conditions used for PCR marker genotyping of the INDEL in the 5' regulatory region of *LanFTc1,* an *FT* homologue in narrow‐leafed lupin (*Lupinus angustifolius* L.).
**Table S3a.** A list of SNP (single nucleotide polymorphism) and INDEL (insertion/deletion) polymorphisms in 43 accessions of narrow‐leafed lupin (*Lupinus angustifolius* L.) in the *LanFTc1* genomic region (encompassing 7 kb upstream and 2 kb downsteam of the gene) relative to the Tanjil reference genome (Hane *et al*. 2017).
**Table S3b.** A list of polymorphisms in 42 accessions of narrow‐leafed lupin (*Lupinus angustifolius* L.) within the 1,423 bp sequence of the 5' regulatory region of *LanFTc1*, a *FLOWERING LOCUS T* homologue, which is deleted in the Tanjil reference genome (Hane *et al*. 2017).
**Table S5.** Transcription factor motifs identified in the wild‐type sequence (ku; represented by accession P27255) of *LanFTc1*, the vernalisation responsive *FT* homologue in narrow‐leafed lupin (Lupinus angustifolius L.).
**Table S6.** A list of candidate transcription factor binding site motifs for sequences within the 5’ regulatory region and 5' UTR of *LanFTc1* that are critical for establishing moderate (C_M_) or high (C_H_) levels of de‐repressed LanFTc1 expression during early vegetative growth.
