# Supplementary material for: INDEL variation in the regulatory region of the major flowering time gene LanFTc1 is associated with vernalization response and flowering time in narrow‐leafed lupin (Lupinus angustifolius L.)
Source: Plant Cell Environ. 2018 May 23;42(1):174–87. doi: 10.1111/pce.13320 (PMC7379684; doi:10.1111/pce.13320)
Supplement: Supplementary file 1 — Figure S1. PCR markers to assay four major INDEL variants (0 bp, 1,423 bp, 1,208 bp and 5,162 bp) in the promoter region of LanFTc1, a FLOWERING LOCUS T homologue of narrow‐leafed lupin. [file PCE-42-174-s001.pdf]

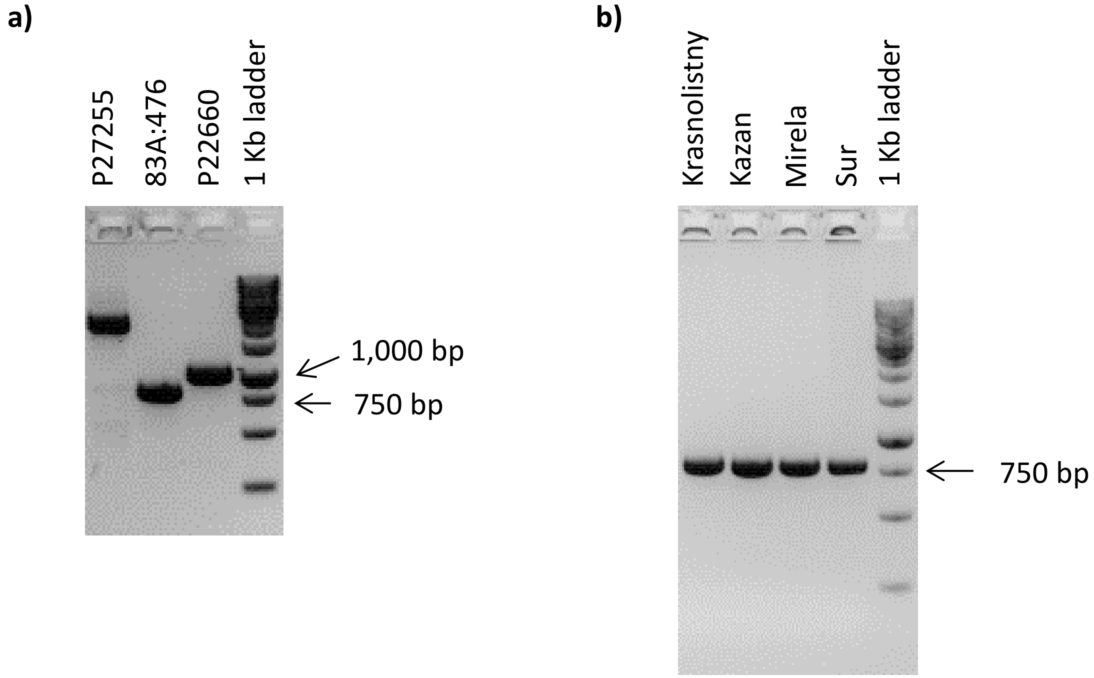

Fig. S1: PCR markers to assay four major INDEL variants (0 bp, 1,423 bp, 1,208 bp and 5,162 bp) in the promoter region of *LanFTc1,* a *FLOWERING LOCUS T* homologue of narrow-leafed lupin.

a) The PCR marker designed by Nelson *et al.* (2017) can assay the 0 bp, 1,423 bp, and 1,208 bp deletions, as demonstrated using the representative accessions P27255, 83A:476, and P22660, which produce amplicons of approximately the expected sizes (2277 bp, 854 bp and 1069 bp, respectively).

b) The PCR marker designed in the current study is able to assay the 5,162 bp deletion, as demonstrated using four representative accessions, Krasnolistny, Kazan, Mirela and Sur, each of which produce amplicons of approximately the expected size (757 bp).
